# Supplementary material for: Tracking Vibrio: population dynamics and ecology of Vibrio parahaemolyticus and V. vulnificus in an Alabama estuary
Source: Microbiol Spectr. 2024 Apr 5;12(5):e03674-23. doi: 10.1128/spectrum.03674-23 (PMC11210274; doi:10.1128/spectrum.03674-23)
Supplement: Supplemental figures and tables. — Figures S1-S6; Tables S1-S3. [file spectrum.03674-23-s0001.pdf]

Supplemental Materials Legend:

Supplemental Figure 1: Rose diagram of wind direction frequency

Supplemental Figure 2: Precipitation and river discharge trends in 2019

Supplemental Figure 3: Nutrient trends at sampling sites in the Eastern Mississippi Sound System (EMSS)

Supplemental Figure 4: Chlorophyll a measured among sites in the EMSS

Supplemental Figure 5: Heat map of phytoplankton occurrence and associated salinity

Supplemental Figure 6: Visualization of low salinity/ high salinity and low turbidity/high turbidity correlated phytoplankton regimes

Supplemental Table 1: Size ranges and HAB status of identified phytoplankton species

Supplemental Table 2: Average water depth and euphotic depths at each sampling site

Supplemental Table 3: PCR mastermix reagents and volumes for *Vibrio parahaemolyticus* and *Vibrio vulnificus* assays

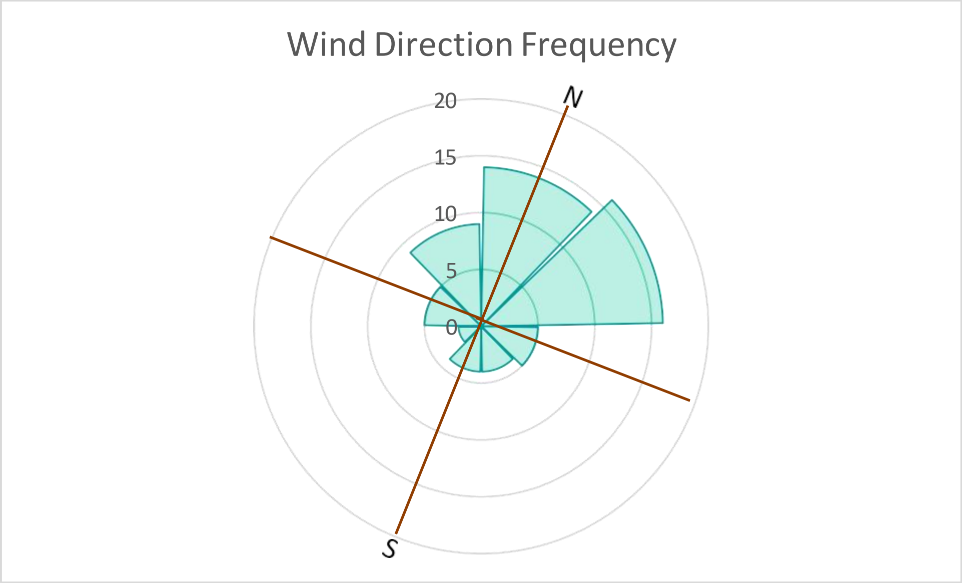

Supplemental Figure 1. Rose diagram of wind direction frequency. This diagram displays in-situ wind direction on sampling days (April-October 2019).

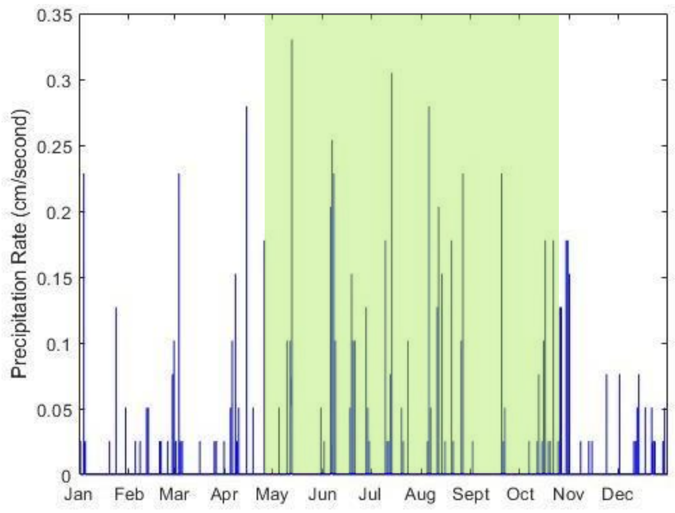

a)

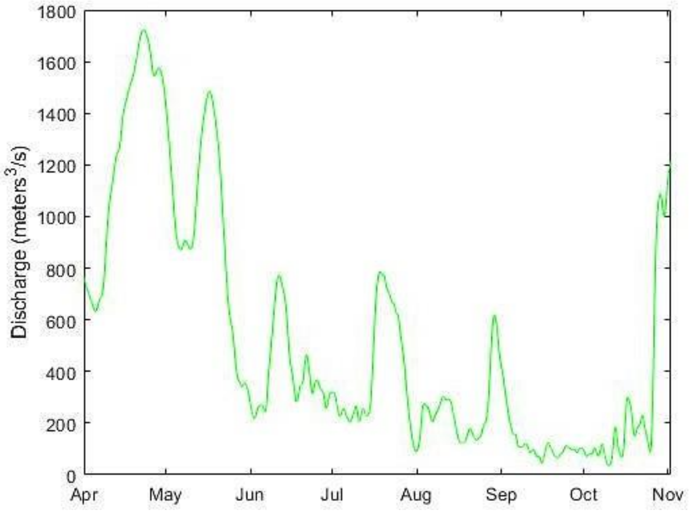

b)

Supplemental Figure 2. Precipitation and river discharge trends in 2019. **a)** Precipitation rate recorded by the Dauphin Island ARCOS meteorological station. **b)** Freshwater discharge rate for the Mobile River during the sampling period, recorded by USGS river gauge station #02470629 in Bucks, AL. The green rectangle indicates the sampling period. Magnitude and intensity of freshwater discharge was mirrored by the Tensaw River as recorded by the USGS river gauge station #02471019 in Mt. Vernon, AL (data not shown). Discharge volumes were filtered to remove the effects of tidal forcing.

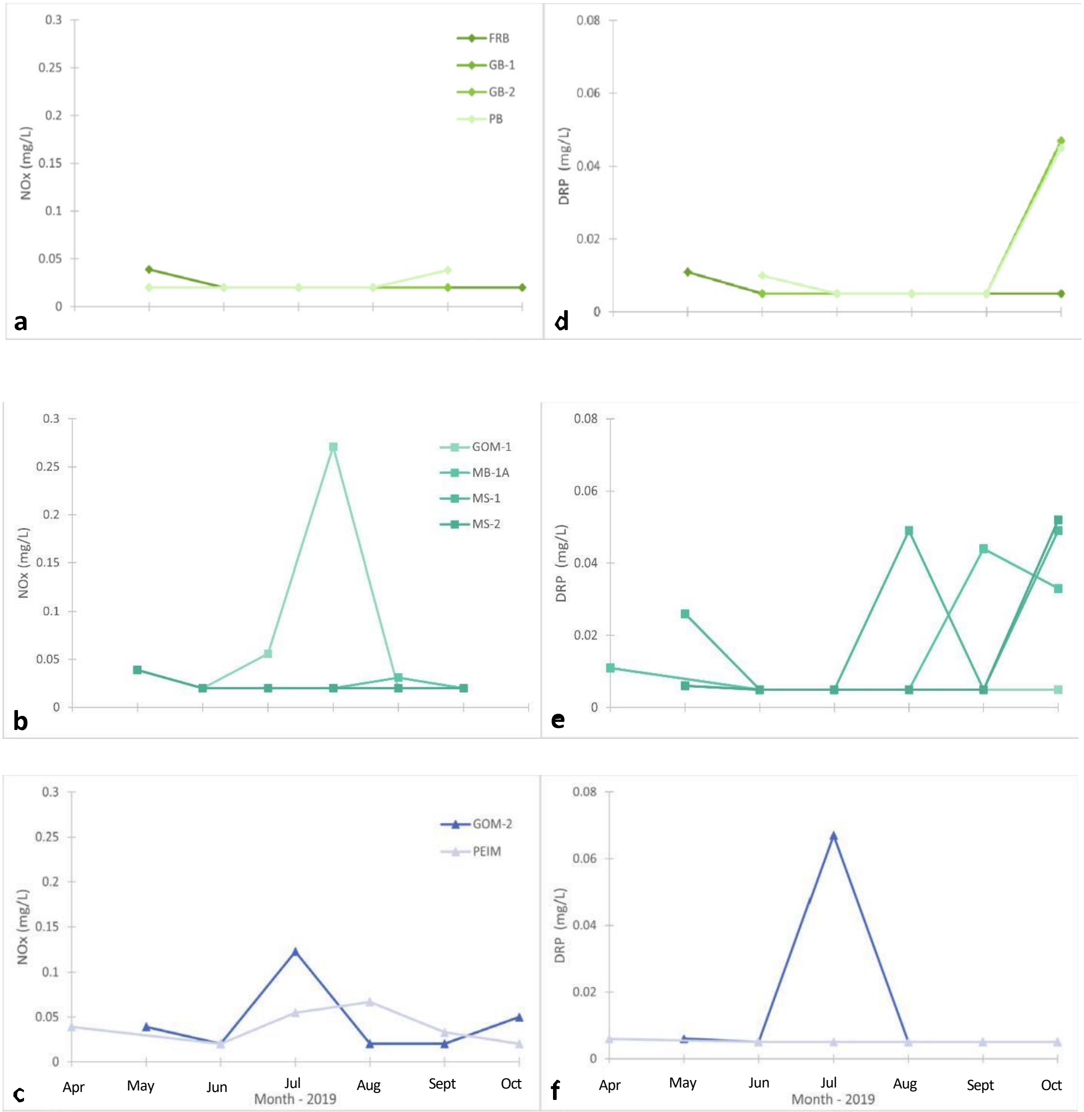

Supplemental Figure 3. Nutrient trends at sampling sites in the Eastern Mississippi Sound System. Nitrate and nitrite concentrations (NOx) are shown in the left column (**a-c**); dissolved reactive phosphorus concentrations are shown in the right (**d-f**). Plots are grouped by region: green indicates coastal bay sites (FRB, GB-1, GB-2, and PB), teal indicates sound sites (MS-1, MS-2, GOM-1, and MB-1A), and blue indicates barrier island sites (GOM-2 and PEIM).

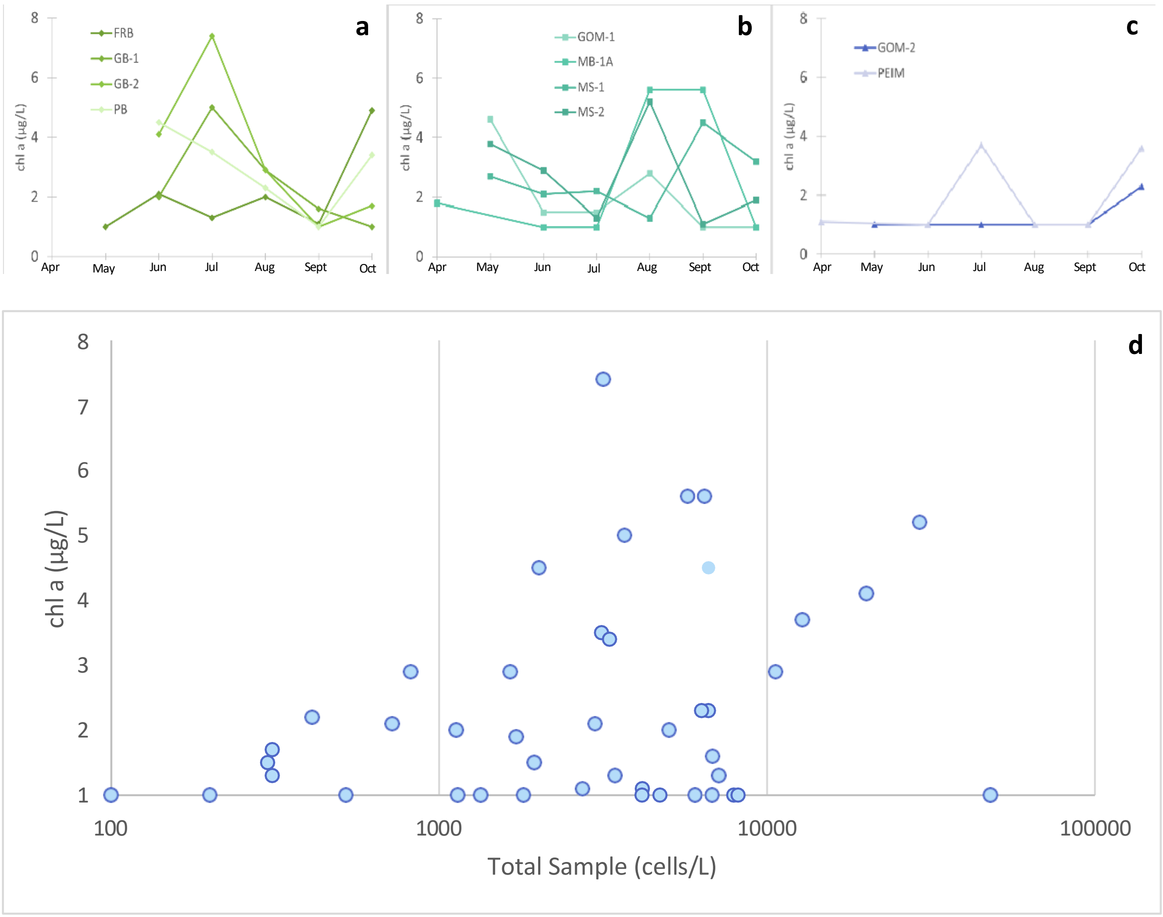

Supplemental Figure 4. a-c) Chlorophyll a measured among sites in the EMSS. Plots in green indicate coastal bay sites, plots in teal indicate sound sites, and plots in blue indicate barrier island sites. **d)** Chlorophyll a concentrations plotted against harmful algal abundances (log transformed). Limit of detection for chlorophyll a was 1 µg/L.

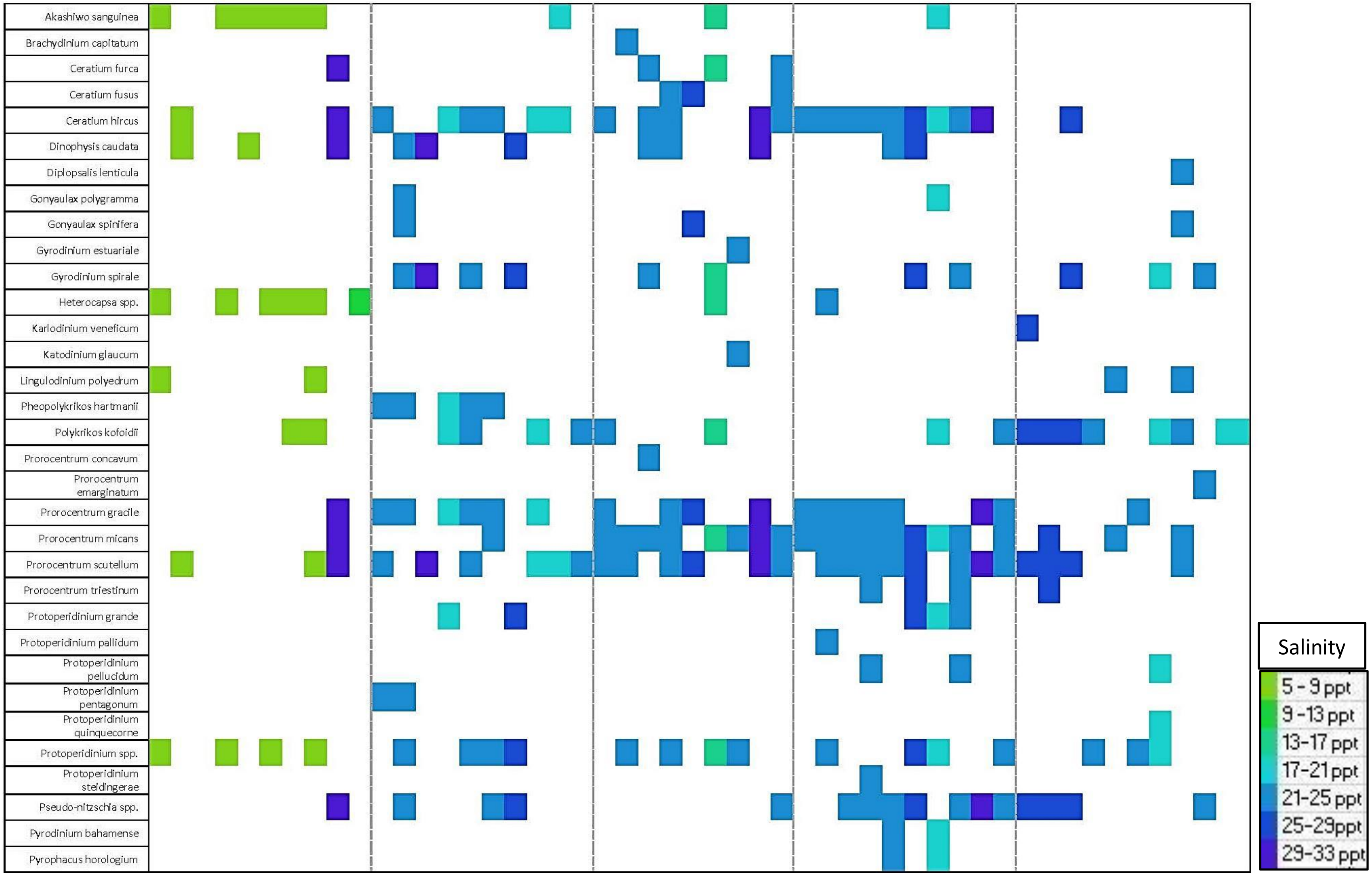

Supplemental Figure 5. Heat map of phytoplankton occurrence and associated salinity. Shaded squares indicate samples where a certain species was recorded; the color of the square signifies the salinity at the site when the species was found.

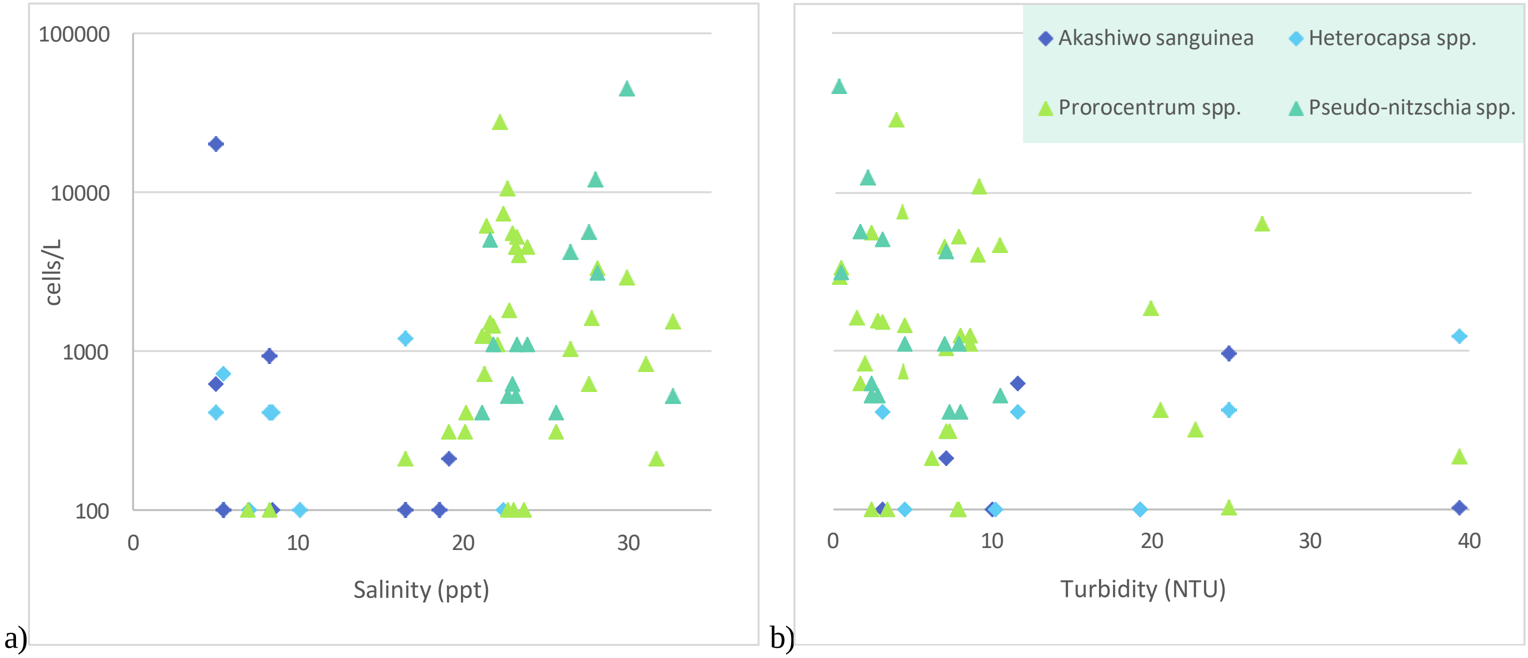

Supplemental Figure 6. Visualization of low salinity/ high salinity and low turbidity/high turbidity correlated phytoplankton regimes. **a)** Plot of salinity and **b)** turbidity with harmful algae concentrations of *Akashiwo sanguinea*, *Heterocapsa* spp., *Prorocentrum* spp., and *Pseudo-nitzschia* spp. Diamonds indicate species associated with lower salinity and triangles indicate species associated with higher salinity.

Supplemental Table 1. Size ranges and HAB status of identified phytoplankton species. The last column on the right indicates which filter size members of this species or genus would likely be caught on in the sequential filtration method outlined in this study. Green highlighted species are those which had significant positive correlations with vibrio abundances. Blue highlighted species are those which had significant negative correlations with vibrio abundances.

Species	Type	Size (µm)	HAB/toxin producer?	Caught on filter
Gyrodinium estuariale	dinoflagellate	9-16		5 µm
Karlodinium veneficum	dinoflagellate	7-18	X	5 µm
Prorocentrum triestinum	dinoflagellate	6-22		5 µm
Ceratium furca	dinoflagellate	30-230	X	35 µm / 5 µm
Ceratium fusus	dinoflagellate	30-231	X	35 µm / 5 µm
Ceratium hircus	dinoflagellate	32-200		35 µm / 5 µm
Diplopsalis lenticula	dinoflagellate	25-70		35 µm / 5 µm
Gonyaulax polygramma	dinoflagellate	26-66		35 µm / 5 µm
Gonyaulax spinifera	dinoflagellate	25-140	X	35 µm / 5 µm
Gyrodinium spirale	dinoflagellate	20-105		35 µm / 5 µm
Heterocapsa spp.	dinoflagellate	9-30		35 µm / 5 µm
Prorocentrum gracile	dinoflagellate	25-55		35 µm / 5 µm
Prorocentrum micans	dinoflagellate	20-75	X	35 µm / 5 µm
Prorocentrum scutellum	dinoflagellate	34-45		35 µm / 5 µm
Protoperidinium quinquecorne	dinoflagellate	30-40		35 µm / 5 µm
Pseudo-nitzschia spp.	diatom	2-175	X	35 µm / 5 µm
Pyrodinium bahamense	dinoflagellate	33-52		35 µm / 5 µm
Pyrophacus horologium	dinoflagellate	30-120		35 µm / 5 µm
Akashiwo sanguinea	dinoflagellate	40-80	X	35 µm
Brachydinium capitatum	dinoflagellate	95-123		35 µm
Dinophysis caudata	dinoflagellate	43-94	X	35 µm
Katodinium glaucum	dinoflagellate	36-62		35 µm
Lingulodinium polyedrum	dinoflagellate	40-60	X	35 µm
Pheopolykrikos hartmanii	dinoflagellate	40-65		35 µm
Polykrikos kofoidii	dinoflagellate	60-160		35 µm
Prorocentrum concavum	dinoflagellate	38-55		35 µm
Prorocentrum emarginatum	dinoflagellate	35-42		35 µm
Protoperidinium grande	dinoflagellate	65-100		35 µm
Protoperidinium pallidum	dinoflagellate	65-100		35 µm
Protoperidinium pellucidum	dinoflagellate	35-52		35 µm
Protoperidinium pentagonum	dinoflagellate	60-80		35 µm
Protoperidinium spp.	dinoflagellate	40-70		35 µm
Protoperidinium steidingerae	dinoflagellate	65-130		35 µm

Supplemental Table 2. Average water depth[1] and euphotic depths at each sampling site.

Site	Average Depth (m)	April Euphotic Depth (m)	May Euphotic Depth (m)	June Euphotic Depth (m)	July Euphotic Depth (m)	August Euphotic Depth (m)	September Euphotic Depth (m)	October Euphotic Depth (m)
FRB	1.3		1.1	1.5	1.5	1.4	1.5	1.3
PB	2.5			2.2	1.7	1.1	2.5	2
GB-1	2.9			2.4	2.7	2.8	2.8	2.8
GB-2	3.1			2.9	2.7	3	3	3.6
MB-1A	3.9	2.9		2.3	1.7	1.5	3.4	2.1
MS-1	2.4		2.2	1.1	2.3	2	2.2	2.4
MS-2	4.6		3.2	4.4	3.4	4.2	4.5	3.8
GOM-1	6		4.8	3.7	5.4	5.5	4.8	4.3
GOM-2	11.5		9	8.7	7.8	10	12	11.2
PEIM	9.9	8		9	5.6	7	10.9	3.1

[1] Entire water column depth was averaged across all sampling months for each site; water column depth varied across the sampling period due to tidal differences, wind forcing, and freshwater input. Therefore, some euphotic depths listed for individual months may be greater than the average depth for the site.

Supplemental Table 3. PCR mastermix reagents and volumes for *Vibrio parahaemolyticus* and *Vibrio vulnificus* assays. JOE is 2′,7′-dimethoxy- 4′,5′-dichloro-6-carboxyfluorescein. Cy5 is a fluorescent cyanine dye. BHQ1 and BHQ2 are Black Hole Quenchers 1 and 2, respectively. *V. vulnificus* primer sequences were originally reported by Campbell and Wright 2003 (96), and *V. parahaemolyticus* primer sequences were reported by Nordstrom et al.(84). *Vibrio vulnificus* cycling protocol started with 1-minute at 95°C followed by 45 cycles of 15 s at 95°C, 15 s at 57°C, and 25 s at 72°C. *Vibrio parahaemolyticus* cycling protocol started with 1-minute at 95°C followed by 45 cycles of 5 s at 95°C and 45 s at 57°C. Data analysis used default analysis parameters, except the manual threshold was changed to 0.02 and the background end cycle was set to 10 for all targets. Cycling and analysis conditions were as described in (85)

Component	Details/ Sequences (5' to 3')	Source	Final Concentration	Used in Vp mastermix	Used in Vv mastermix
PCR Water		Invitrogen	-	12.765 µL/rxn	12.220 µL/rxn
PCR Buffer		Invitrogen	1 X	X	X
$MgCl_2$	50 mM stock	Invitrogen	5.0 mM	X	X
dNTPs	Mixed equal concentrations of each	Roche, Indianapolis, IN	0.3 mM	X	X
tlh 884 F forward primer	ACTCAACACAAGAAGAGATCGACCA	Integrated DNA Technologies (IDT), Coralville IA	0.2 µM	X	
tlh 1091 R reverse primer	GATGAGCGGTTGATGTCCAA	IDT	0.2 µM	X	
vvh forward primer	TGTTTATGGTGAGAACGGTGACA	IDT	0.3 µM		X
vvh reverse primer	TTCTTTATCTAGGCCCCAAACTTG	IDT	0.3 µM		X
IAC 46 F forward primer	GACATCGATATGGGTGCCG	IDT	0.08 µM	X	X
IAC 186 R reverse primer	CGAGACGATGCAGCCATTC	IDT	0.08 µM	X	X
tlh probe	CGCTCGCGTTCACGAAACCGT Modifications: 5' JOE – 3' BHQ2	IDT	0.15 µM	X	
vvh probe	CCGTTAACCGAACCACCCGCAA Modifications: 5' Cy5 – 3' BHQ2	IDT	0.2 µM		X
IAC Cy5 probe	TCTCATGCGTCTCCCTGGTGAATGTG Modifications: 5' Cy5 - 3' BHQ2	IDT	0.15 µM	X	
IAC JOE probe	CGCTCGCGTTCACGAAACCGT Modifications: 5' JOE – 3'BHQ1	IDT	0.15 µM		X
Taq Polymerase	U Platinum *Taq* DNA Polymerase	Invitrogen	Vp- 1.5 unit/µL Vv- 1.2 unit/µL	0.30 µL/rxn	0.22 µL/rxn
Passive Reference Dye	ROX	ThermoFisher, Waltham, MA	-	X	X
Internal Amplification Control DNA (IAC)		Patent referenced in Nordstrom et al. (84)	-	X	X

(96). Campbell MS, Wright AC. 2003. Real-time PCR analysis of *Vibrio vulnificus* from oysters. Appl Environ Microbiol 69:7137–7144. https://doi.org/10.1128/AEM.69.12.7137-7144.2003
